# Multisegment Foot Kinematics During Walking in Younger and Older Adults

**DOI:** 10.4021/jocmr984w

**Published:** 2012-07-20

**Authors:** Dominique Legault-Moore, Victoria L. Chester, Gwyneth de Vries

**Affiliations:** a Andrew and Marjorie McCain Human Performance Laboratory, Richard J. Currie Center, Faculty of Kinesiology, University of New Brunswick, Fredericton, NB, E3B 5A3, Canada; b Department of Orthopaedic Surgery, Dr. Everett Chalmers Hospital, Fredericton, New Brunswick, E3B 6H5, Canada

**Keywords:** Gait, Multisegment foot, Aging, Kinematics

## Abstract

**Background:**

Currently, age-related changes in foot mechanics are poorly understood. A greater understanding of the natural changes in foot motion is needed to improve our understanding of pathological foot conditions.

**Methods:**

The purpose of this study was to compare multisegment foot kinematic data during gait in younger and older individuals. Eleven (N = 11) adult male participants between the ages of 18 - 30 years (younger group; mean ± SD: 24.6 ± 3.0 years) and eleven (N = 11) adults aged 55 years or older (older group; mean ± SD: 65.0 ± 4.2 years) were recruited for the study. The foot was modeled as a four-segment rigid body model. Three-dimensional kinematic and kinetic gait parameters were recorded using an 8-camera Vicon MCam motion capture system and two Kistler force plates. A MANOVA was used to test for significant differences in mean temporal-spatial data, mean ranges of motion, and mean peak joint angle data between age groups.

**Results:**

No significant differences (P > 0.05) were found between the two age groups for any of the gait parameters. The results of the present study suggest that individuals aged 65.0 ± 4.2 years have foot mechanics that are comparable to younger walkers.

**Conclusions:**

As such, any deviations in motion at this age may be indicative of an underlying disease or disorder.

## Introduction

The foot plays a critical role during gait, providing a base of support and aiding in shock absorption and propulsion. To date, the majority of foot biomechanics research has modeled the foot as a single rigid body. Over the last decade, there has been an increase in the number of studies that have used multisegment foot models [1-4]. These models have increased our knowledge of the complex movements within the foot during gait. Such models are beneficial for research examining the relationship between foot function and aging, disease, and treatment efficacy.

Previous research has suggested that changes in foot structure and function occur with age. Changes include decreased range of joint motion [5-9], decreased strength [5, 8-9], lowering of the transverse arch [9-10], and decreased proprioception [11]. Such changes may lead to alterations in foot mechanics and could have important consequences to an individual’s mobility and quality of life.

Gait studies that compared the foot biomechanics of healthy younger and older populations have typically used single rigid segment models. Decreased ankle plantarflexion [12-15], decreased ankle joint range of motion [16], reduced peak ankle joint power [14, 17-18], and a more flat-footed landing [14], have been reported for older walkers. However, research using more complex multisegment models of the foot in the aging population is limited. The majority of studies examining healthy feet using multisegment models have typically assessed children to adults [1-4, 19-24]. Studies that included individuals aged 55 years or older have combined the data for the older participants with younger participants [19, 23]. Therefore, we currently know very litte about the multisegment foot mechanics in older adults specifically. Multisegment foot motion could provide additional insight into age-related changes in foot mechanics in older populations.

To our knowledge, no studies have compared multisegment foot kinematics in younger and older age groups. Research that examines multisegment foot kinematics across age spans is needed to increase our knowledge of age-related changes in foot movement. A greater understanding of these naturally occurring changes could play an important role in identifying foot/ankle pathology in older populations. Therefore, the purpose of this study is to compare multisegment foot kinematic data during gait in younger and older individuals.

## Method

### Participants

Twenty-two (N = 22) adult male participants between the ages of 18-30 years (younger group; N = 11) and 55 years or older (older group; N = 11) were recruited for the study. Further characteristics of each group are provided in Table 1. With the exception of age, there were no significant differences (P > 0.05) between the old and young groups for mass, height, or shoe size. All participants had a minimum shoe size of nine to facilitate marker tracking and autolabeling. Female participants were excluded from the study for two reasons: 1) on average, females have smaller feet than males, which causes an increase in the incidence of marker merging and tracking problems; and 2) a sample of only males eliminates any gender effects that may be associated with foot kinematics. A medical history questionnaire was completed by each individual. Individuals with a history of diseases/disorders that could affect foot kinematics, including diabetes, neurological pathologies, joint replacement surgeries, chronic pain, and edema were excluded from the study. Participants were recruited through advertisements, emails, and word-of-mouth. This study was approved by the University Research Ethics Board.

**Table 1:** Participant Characteristics (Mean ± 1 SD) for the Older and Younger Groups

	Younger		Older	
	Mean	SD	Mean	SD
Age (years)	24.6	3.0	65.0	4.2
Height (cm)	178.5	5.8	174.6	6.3
Weight (kg)	79.5	9.7	78.7	14.6
Foot Size	10.3	1.1	9.8	1.1

### Instrumentation

An eight camera Vicon MCam motion capture system (Oxford Metrics Group, Oxford, UK), sampling at 120 Hz, was used to track the three-dimensional trajectories of reflective markers (diameter of 14 and 25 mm) placed on the participant's skin. Two force plates (Kistler 9281CA, Kistler Instruments, Winterthur, Switzerland), embedded and disguised in the lab floor, were used to aid in the identification of gait cycle events. A weight scale, measuring tape, and calipers were used to obtain anthropometric measures from each subject.

### Procedures

Fifteen (N = 15) reflective markers were placed directly on the right foot and tibia of each participant (Table 2). For consistency, the same researcher was always responsible for placing markers on each participant in the study. Following this, a static capture of the participant during quiet standing in the anatomical position was recorded to permit the calculation of offset values for all joint rotations. These joint offset values were later subtracted from the appropriate joint rotations for the gait cycles of each participant. This offset calculation did not apply to the planar angles. Following the static trial, each participant was asked to walk barefoot across the lab at a self-selected speed. Several practice trials were completed to allow the participants to adjust to the markers and the lab environment. Participants were then asked to perform at least 6 successful right limb gait cycles (e.g. clean force plate strike and marker visibility) at a self-selected speed.

**Table 2:** Anatomical Landmarks for the Four-Segment Model of the Foot and Shank

Segment	Location
Hallux	Most distal and dorsal point of the head of the proximal phalanx
Forefoot	Head of the fifth metatarsal, dorso-lateral aspect of the fifth metatarso-phalangeal joint
	Head of the second metatarsal, dorso-medial aspect of the second metatarso-phalangeal joint
	Head of the first metatarsal, dorso-medial aspect of the first metatarso-phalangeal joint
	Base of the fifth metatarsal, dorso-lateral aspect of the fifth metatarso-cuboid joint
	Base of the second metatarsal, dorso-medial aspect of the second metatarso-cuneiform joint
	Base of the first metatarsal, dorso-medial aspect of the first metatarso-cuneiform joint
Calcaneus	Lateral calcaneus - midpoint between the lateral malleolus and floor
	Medial calcaneus - point between medial malleolus and floor at height of lateral calcaneus marker
	Posterior calcaneus - aligned vertically with Achilles tendon at same height as lateral calcaneus marker
Shank	Most lateral aspect of lateral malleolus
	Most medial aspect of the medial malleolus
	Most lateral prominence of lateral epicondyle
	Most medial prominence of the medial epicondyle
	Most anterior aspect of the tibial tuberosity

### Multisegment foot model

**Figure 1:**
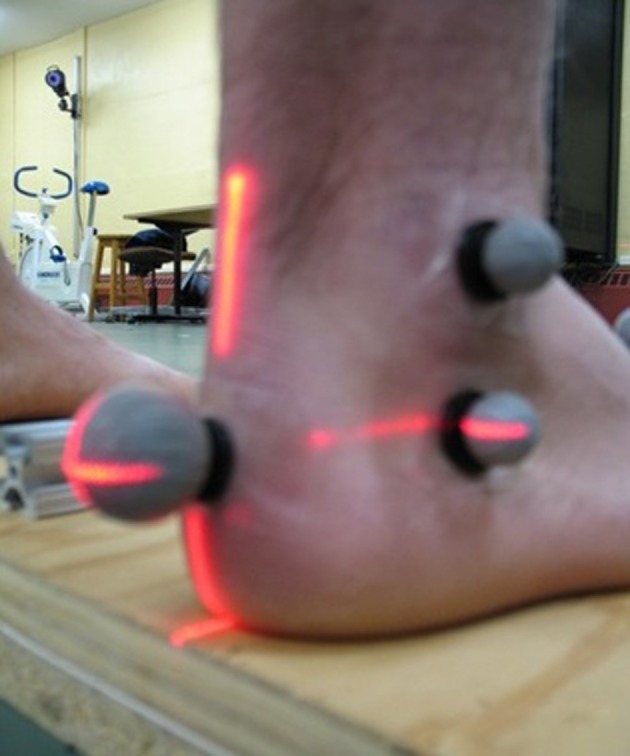
Laser technique used to align calcaneus markers.

Four foot segments were created and assumed to be rigid: 1) the shank; 2) the total foot (single rigid segment); 3) the calcaneus; and 4) the forefoot (included all 5 metatarsal bones). The hallux and metatarsal bones were modeled as line segments for the computation of planar angles. The anatomical landmarks (Table 2) and reference frames were consistent with the model developed by Leardini et al [1], with the following exceptions: 1) the midfoot segment was excluded from the present study due to difficulties with consistent tracking of the navicular and cuboid markers; and 2) in contrast to Leardini et al [1], a neutral calcaneus was formed using a laser level technique to guide marker placement (Fig. 1). This ensured the reliable placement of markers on a segment with few palpable anatomical landmarks. To achieve a neutral calcaneus, the vertical midpoint between the floor and the lateral malleolus was determined using calipers. A marker was placed at this point and referred to as the lateral calcaneus. A cross-hair laser was then used to aid in the placement of the posterior calcaneus marker. The vertical laser line was aligned with the midsection of the Achilles tendon, while the horizontal laser line was aligned with the lateral calcaneus marker. The intersection of the two laser lines formed the location for the posterior calcaneus marker. Lastly, using the laser level, the medial calcaneus marker was placed beneath the medial malleolus at the same height as the lateral and posterior calcaneus markers.

### Data analysis

Data was analyzed using custom software created in Matlab (Mathworks, Inc). For each participant, trial selection involved the computation of cadence, velocity, and percent of cycle spent in single stance for each successful gait cycle. The single gait cycle that most closely approximated the individual mean of all gait cycles on these three measures was selected as the single trial for analysis for each participant. The locations of the three non-collinear markers on each rigid segment were used to create embedded coordinate systems at the virtual joint centers [1]. Joint angles were computed from the relative orientations of the embedded coordinate systems using Euler angles in an yxz sequence, corresponding to flexion/extension, adduction/abduction, and internal/external rotation. Displacement data were filtered using a zero phase lag, second order Butterworth filter with a cutoff frequency of 6 Hz. Joint angle data were normalized to 100% of the gait cycle. A MANOVA was used to test for significant differences in mean temporal-spatial data (Table 3), mean range of motion, and mean peak joint angle data between age groups (Table 4).

**Table 3:** Descriptive Data (Mean ± 1 S.D.) for Temporal-Spatial Variables for the Younger and Older Age Groups

Temporal-spatial Measure	Younger (N = 11)		Older (N = 11)	
	Mean	SD	Mean	SD
Cycle Time (s)	1.1	0.1	1.1	0.1
Cadence (steps/min)	106.2	4.6	107.6	8.7
Stride Length (m)	1.4	0.1	1.4	0.2
Walk Speed (m/s)	1.2	0.1	1.2	0.2
Opposite Toe-off (%)	12	1.5	11.5	1.7
Opposite Foot Strike (%)	49.6	1.5	50.1	1.6
Toe-off (%)	61.3	1.3	60.7	1.6
Single Stance (%)	37.6	1.2	38.6	2
Double Stance (%)	23.6	2.2	22	2.8
Stance (%)	61.3	1.3	60.7	1.6

**Table 4:** Descriptive Results (Mean ± 1 S.D.) for Joint Angles for the Younger and Older Groups During the Stance Phase, Swing Phase, and Entire Gait Cycle

Variables	Phase	Young Group (N = 11)		Old Group (N = 11)	
		Mean (º)	SD	Mean (º)	SD
Hindfoot - Shank					
Max inversion	Cycle	-8.00	5.20	-7.40	3.20
Max eversion	Cycle	4.50	3.00	4.70	1.80
Eversion ROM	Cycle	12.50	3.50	12.10	3.00
Max plantarflexion	Stance	-11.30	2.20	-12.20	3.90
Max plantarflexion	Swing	-14.80	6.40	-12.20	8.00
Max dorsiflexion	Cycle	8.50	2.40	9.20	3.30
Dorsiflexion ROM	Cycle	19.80	2.10	21.40	2.40
Max abduction	Cycle	-10.30	2.60	-9.80	4.20
Max abduction	Cycle	3.80	2.70	5.00	2.10
Abduction ROM	Cycle	14.10	2.90	14.70	3.60
Hindfoot - Forefoot					
Max eversion	Cycle	8.40	3.50	9.10	2.00
Max plantarflexion	Cycle	-13.50	3.90	-11.70	4.10
Max dorsiflexion	Cycle	3.70	3.40	4.40	3.10
Dorsiflexion ROM	Cycle	17.20	5.60	16.10	3.80
Foot - Shank					
Max inversion	Cycle	-8.80	3.30	-12.00	4.20
Max plantarflexion	Stance	-12.40	5.10	-10.60	5.00
Max plantarflexion	Swing	-17.40	7.30	-14.60	4.30
Max dorsiflexion	Cycle	12.30	2.30	12.40	4.50
Dorsiflexion ROM	Cycle	24.70	4.60	23.00	3.60
Planar Angles [1]					
V2G Max	Cycle	74.10	11.80	68.30	12.50
S2G Max	Cycle	107.40	10.00	102.60	10.90
F2Ps Max	Cycle	45.50	3.80	41.80	8.60
MLA Max	Cycle	171.50	7.30	171.20	6.90

## Results

**Figure 2:**
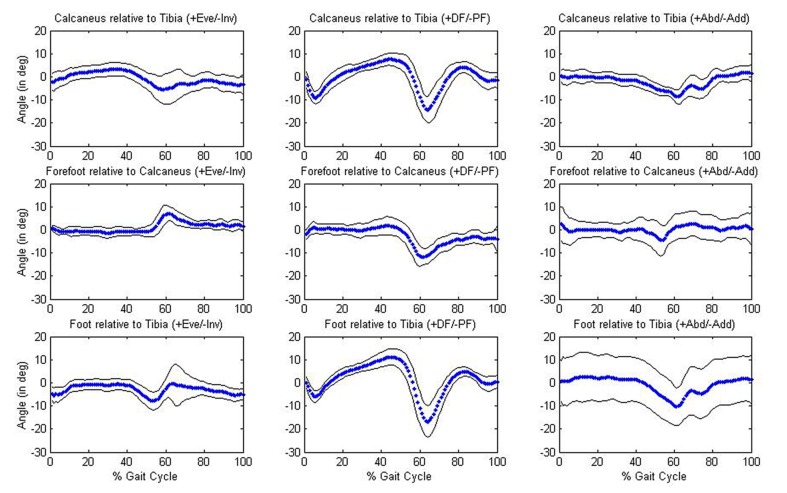
Joint angles for the younger group. Graphs in the first row of figure depict the calcaneus relative to the tibia; graphs in the second row depict the forefoot relative to the calcaneus; graphs in the third row depict the whole foot relative to the tibia.

**Figure 3:**
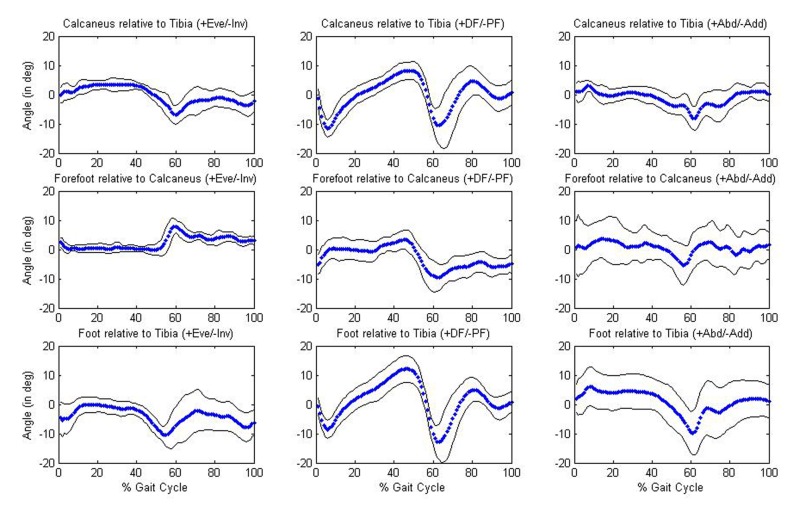
Joint angles for the older group. Graphs in the first row of figure depict the calcaneus relative to the tibia; graphs in the second row depict the forefoot relative to the calcaneus; graphs in the third row depict the whole foot relative to the tibia.

Significant (P < 0.05) differences in kinematic parameters between the younger and older age groups were tested using a MANOVA. No significant differences (P > 0.05) in mean temporal-spatial or mean joint angle parameters were found between age groups. Descriptive data for the temporal-spatial and joint angle data for each group are provided in Tables 3-4. Overall, mean temporal-spatial and mean joint angle parameters were very similar across the two groups. Of particular importance was that mean walking speed was very comparable in the young (1.20 m/s) and old (1.22 m/s) groups. The older group showed slightly larger variability across variables. Figure 2 and Figure 3 provide graphical results of the joint angles examined.

## Discussion

The present study examined age-related differences in multisegment foot kinematics during gait. No significant differences (P > 0.05) were found between the younger and older age groups for the mean temporal-spatial or mean joint angle data. In addition, no significant differences (P > 0.05) were found in height, mass, and shoe size measures between groups. As previous studies have not examined age-related changes in multisegment foot kinematics, comparisons of data across studies were not possible. However, for both the younger and older group, multisegment foot kinematic data was similar to previously published control data from numerous studies [1, 20-26].

Previous gait studies using single rigid segment models of the foot have reported varying results for ankle angles as a function of age. For example, Judge et al [13] found decreased plantarflexion in late stance in a group of older walkers (mean age: 79 years; range: 70 - 90 years) compared to younger walkers (mean age: 26 years; range: 18 - 42 years). The more advanced age of the older walkers in Judge et al [13] versus the present study may account for the differential results for the ankle joint. However, DeVita and Hortobagyi [27] also found decreased plantarflexion for older walkers (mean ± SD: 69 ± 6.5 years) compared to younger participants (mean ± SD: 21.6 ± 2.7 years) who were similar in age to the present study. In contrast, Ostrosky and vanSwearingen [28] found no significant differences in ankle plantarflexion between older (mean: 67.4 years; range: 60 - 80 years) and younger (mean: 28.2 years; range: 22 - 39 years) walkers.

Previous research has also reported decreased ankle range of motion during walking in older walkers versus younger walkers. Hageman and Blanke [16] reported decreased ankle range of motion in older women (mean: 66.85 years; range: 60.0 - 84.0 years) compared to younger women (mean: 23.92 years; range: 20.0 - 33.0 years) while walking at a self-selected speed. These differences were hypothesized to be related to the slower walking velocity of the older group. However, Kerrigan et al [18] reported decreased plantarflexion angles in the elderly (mean: 72.7 years; range: 65 - 84 years) at walking speeds that were both faster and slower than the comfortable walking speed of a young control group (mean: 28.5 years; range: 18 - 36 years). In the present study, mean self-selected walking velocities were similar for the younger and older groups and no significant differences were found in ankle range of motion.

Similar foot mechanics between age groups has important implications for clinicians assessing foot pathology in older adults. Research to date has suggested that foot function changes with age. The results of the present study suggest that individuals aged 65.0 ± 4.2 years have typical foot mechanics during gait. Therefore, deviations in motion at this age may be indicative of an underlying disease or disorder.

A limitation of this study includes the small sample size of both the younger and older age groups (N = 11 for each group). Additionally, while individuals aged 55 years or older were sought for this study, the recruited older participants formed a group with a narrow age range. Therefore, a cross-section of the elderly population was not achieved. The results of this study are specific to the age range and gender of the individuals that participated.

### Conclusions

This is the first study to provide a comparison of younger and older multisegment foot kinematics during gait. A greater understanding of the natural changes in foot mechanics with aging will allow clinicians to more accurately identify foot pathology. The lack of significant differences between the two age groups suggests that normal foot mechanics are present in older individuals. Abnormal foot mechanics as a function of age should not be assumed. Future work will focus on repeating the study with larger sample sizes to validate the findings. In addition, female participants and samples with wider age ranges will be included.
